# Non-Invasive Analyses of Altered Schaedler Flora in C57Bl/6J and Balb/c Mice to Monitor Hygiene Status of a Housing Facility

**DOI:** 10.3390/ani15121725

**Published:** 2025-06-11

**Authors:** Rebecca Nistelberger, Patrizia Gibler, Lisa Barones, Arno Absenger, Julia B. Kral-Pointner, Manuel Salzmann, Boris Hartmann, Bruno K. Podesser, Phillip J. Hohensinner, Roberto Plasenzotti

**Affiliations:** 1 Medical University of Vienna Core Facility, Medical University of Vienna, 1090 Vienna, Austria; rebecca.nistelberger@medunwien.ac.at; 2 Ludwig Boltzmann Institute for Cardiovascular Research, 1090 Vienna, Austria; patrizia.gibler@meduniwien.ac.at (P.G.); bruno.podesser@meduniwien.ac.at (B.K.P.); 3 Biomedical Research, BMF, Medical University Graz, 8010 Graz, Austria; lisa.barones@medunigraz.ac.at (L.B.); arno.absenger@medunigraz.at (A.A.); 4 Internal Medicine II, Medical University of Vienna, 1090 Vienna, Austriamanuel.salzmann@meduniwien.ac.at (M.S.); 5 Institute of Veterinary Disease Control, AGES, 2340 Moedling, Austria; boris.hartmann@ages.at; 6 Center for Biomedical Research, Medical University of Vienna, 1090 Vienna, Austria; 7 Austrian 3R Center, 8020 Graz, Austria; 8 SAN Group GmbH, 3130 Herzogenburg, Austria

**Keywords:** microbiome, laboratory animal housing, altered Schaedler flora, hygiene status, FELASA recommendations

## Abstract

The composition of the gut microbiome, defined by environmental factors, significantly affects research outcomes, with variations observed across animal facilities. Efforts to standardize led to the definition of the ‘Altered Schaedler flora’ (ASF), comprising eight bacterial groups. Our data highlights the variability of ASF under pathogen contact. Feces from two wild-type strains (*C57Bl/6J* and *Balb/c* mice) with and without proven infection was collected in two different animal facilities and analyzed. The data show a significant difference in the quantity (either reduction or increase) of the eight ASF bacterial groups when comparing infected and non-infected mice across different housing areas (SPF-specific pathogen-free, quarantine, and conventional-experimental areas) within a facility, as well as in comparison to another facility. Furthermore, strain-specific differences are also evident, with certain ASF groups showing a reduction in quantity at one facility but an increase at the other, comparing the same housing area. Comparative studies across facilities confirmed the necessity of baseline determination for accurate ASF analysis. Performing ASF analysis, facilitated by in-house qPCR (quantitative polymerase chain reaction) kits, offers prompt and precise microbiome profiling, enhancing experimental accuracy and health monitoring in animal research settings.

## Simple Summary

Transgenic animal models are crucial for precise genetic research. The gut microbiota plays a vital role, affecting digestion, metabolism, immune development, and pathogen exclusion. The microbiome’s composition varies with environmental factors and can significantly impact research outcomes, necessitating standardization for further microbiome research. The ‘Altered Schaedler flora’ (ASF), consisting of eight bacterial groups, was defined after analyzing the whole gut microbiome of laboratory mice. These ASF models, showing stability across generations, are used to establish baseline microbiome conditions to simplify comparability, as only a relatively small data set is available (many groups of bacteria tend to constantly change in composition and occurrence). Our data show a significant difference in the quantity of ASF groups when comparing laboratory mice held in specific pathogen-free housing conditions to mice housed in areas with proven pathogen contact. It is also interesting to note that there were strain-specific differences between the two mouse lines tested. In conclusion, it can be said that an in-house ASF analysis is a good tool for detecting an unwanted infection. However, a baseline must first be defined for the respective animal strain and husbandry area.

## 1. Introduction

In contemporary research, transgenic animal models are frequently utilized. Through targeted genetic modifications, desired genotypes and phenotypes can be generated, enabling more precise and extensive research investigations. An often-overlooked factor in these studies is the presence of specific pathogens that interfere with research goals. Therefore, animal husbandry conditions need to be optimized to minimize this risk [1]. To detect unwanted changes, the composition of the gut microbiome, which is in constant exchange with its host, has proven to be a valuable analysis tool [2]. The microbiota comprises all symbiotically growing microorganisms within a living being [3]. The gut microbiota contributes to digestive processes and modulates metabolism, and it is influenced by various environmental factors [4,5,6].

It has been demonstrated that there is a close link between the host and its gut microbiome. Healthy individuals benefit from the gut microbiome through the production of metabolic products, the development of the immune and enteric nervous systems, and the competitive exclusion of pathogens harmful to the host [7,8,9]. Moreover, the gut microbiome influences outcomes in animal studies [10], and its composition impacts test results, varying significantly across animal facilities [11].

Due to its enormous number of bacteria, efforts have been made to simplify the overview. In the 1970s, eight common bacterial groups, named ‘Altered Schaedler flora’ (ASF), were defined and established by R.P. Orcutt [12], following the principles of R.W. Schaedler, to facilitate the investigation of host–microbiota relationships [12]. This led to the creation of mice with a standardized intestinal microbiome by inoculating germ-free mice with defined bacterial groups [13]. The selection criteria for these groups were based on consistency across generations in commonly housed laboratory mice and the absence of morphological changes in the digestive tract, resembling normal mice [14,15]. Research has shown that the eight ASF strains functionally represent wild gut microbiomes better compared to the genetic content of ASF in wild murine metagenomes [16] and can be used as an analytical tool to evaluate environmental influences in animal housing conditions [14]. Highlighting the importance of ASF in research, Charles River Laboratories and Taconic Biosciences use ASF to establish baseline conditions in the gut microbiome when starting their colonies [17,18].

To enhance the representability of ASF, Wannemuehler et al. [9] performed whole-genome sequencing to generate draft sequences of the eight ASF bacterial groups. These analyses can demonstrate how the intestinal microbiome responds to diet changes, genetic background variations, or pathogenic infections. Furthermore, Sarma-Rupavtarm et al. [15] developed protocols for the quantification of ASF strains using 16S RNA analyses, and ready-to-use primer kits are now available for qPCR of altered Schaedler flora (Section 2, pp. 3–4).

Although ASF can be cultivated and shows great stability over several generations, strain-specific differences in its composition have been observed [14,19]. Nevertheless, ASF represents all relevant bacterial niches of the GI tract and serves as sentinel flora to better represent environmental influences, which can be crucial in experimental settings [14]. Pathogen contact can also impact ASF composition and interaction [7]. Typically, unwanted pathogen contact is detected via direct pathogen detection using PCR at regular intervals in larger animal facilities. Many facilities follow the recommendations of the Federation of European Laboratory Animal Science Associations (FELASA), which plays a significant role in modern laboratory animal practice [20].

Currently, there is limited literature on changes in ASF upon pathogen contact. We propose that ASF composition changes upon contact with unwanted FELASA-relevant pathogens in SPF laboratory animal housing areas [20], which can be visualized by qPCR, reflecting the health status of a housing facility.

## 2. Materials and Methods

Mice (in-house, purchased from Janvier, Le genest st isle, France) were housed in IVC (individual ventilated) cages (Tecniplast; Tecniplast Group, Varese, Italy) with aspen wood bedding (Las Vendi, Soest, Germany), standard mouse food (Altromin, Lage, Germany), and autoclaved drinking water, according to Austrian Animal Experimentation Law. The room temperature was maintained at 21 °C (±2 °C) with a 55% (±10%) humidity level and a 12 h day/night light cycle. Sampling was conducted by employees of the respective housing area. A PCR for FELASA-recommended pathogens [20] was performed by a company specialized in health monitoring of laboratory animals.

Mice from the external facility (derived from Charles River, Sulzfeld, Germany) were housed in IVC cages (Tecniplast, Tecniplast Group, Varese, Italy) with aspen wood bedding (Mucedola, Milan, Italy) in the SPF area and aspen wood bedding (Rettenmaier und Söhne GmbH, Rosenberg, Germany) in the quarantine area. In the SPF area, mice were provided with autoclaved drinking water and the same food (Sniff and Altromin, Soest, Germany). In the quarantine area, no autoclaved drinking water was used. The room temperature was 21 °C (±2 °C) with a 55% (±10%) humidity level and a 12 h day/night light cycle.

### 2.1. Feces from Cages Housing C57Bl/6J and Balb/c Mice Were Collected in Three Different Settings

#### 2.1.1. Setting 1 (ASF Analysis in Balb/c and C57Bl/6J in Controlled Infection)

C57Bl/6J and Balb/c mice (8 mice per group, 8–9 weeks old) were inoculated with murine coronavirus MCoV MHV-A59. The virus was expanded and isolated as previously published [21]. Mice were inoculated with 20 μL MCoV (1.5 × 10^4^ TCID50) intranasally under general anesthesia. General anesthesia was administered using 0.5 mg/kg medetomidine (Domitor^®^, Orion Pharma, Vienna, Austria) and 5 mg/kg midazolam-Accord (Accord Healthcare, Devon, UK) intraperitoneally. Antagonization was performed with 2.5 mg/kg atipamezole (Antisedan^®^, Orion Pharma, Vienna, Austria) and 0.5 mg/kg flumazenil (Pharmaselect, Vienna, Austria) subcutaneously 15 min after the anesthesia injection. The presence of the virus was detected by in-house qPCR. The control group was housed under the same conditions but was not infected.

Ten fecal pellets from a total of 8 housing cages (4 mice per cage and 32 animals in total) from the C57Bl/6J and Balb/c strains were collected (including infected mice and controls).

#### 2.1.2. Setting 2 (ASF Analysis in Natural Infection Facility 1)

Fifteen fecal pellets from a total of six housing cages (3–5 mice per cage) from the *C57Bl/6J* and *Balb/c* strains (12 cages in total) were collected in the SPF (specific pathogen free) and quarantine housing units of the same facility. The health report of the quarantine unit indicated the presence of MNV, *Pasteurella pneumotropica* (Jawetz), *Pasteurella pneumotropica* (Heyl), *Helicobacter* spp. (*Helicobacter ganmani, Helicobacter mastomyrinus, Helicobacter typhlonius*), and Protozoa (*Tritrichomonas muris, Chilomastix* spp.). The health report of the SPF unit indicated none of the abovementioned or other FELASA-recommended pathogens, which should be avoided because of their known impact on different research aims [22].

#### 2.1.3. Setting 3 (ASF Analysis in Natural Infection in Facility 2)

Fifteen fecal pellets from a total of six housing cages (3–5 mice per cage) from the *C57Bl/6J* and *Balb/c* strains (12 cages in total) were collected in the SPF (specific pathogen free) and quarantine housing units of the same facility. The health report of the quarantine unit indicated the presence of *Mouse Norovirus* (MNV) and *Helicobacter* spp. The health report of the SPF unit indicated none of the abovementioned or other FELASA-recommended pathogens [22].

#### 2.1.4. qPCR and Statistics

Statistic

Normality was tested using Anderson-Darling, or, if the sample size was smaller than 8, the Kolmogorov–Smirnov test was used. Data with Gaussian distribution were checked for statistical significance using Student’s *t*-test with Welch’s correction for unequal SD, and data without Gaussian distribution were analyzed using the Mann–Whitney U-test. All tests were performed using GraphPad Prism 8.

qPCR

The qPCR was performed using a C1000 Touch Thermal Cycler (Bio-Rad, Hercules, CA, USA)) and GoTaq qPCR Master Mix (Promega, Walldorf, Austria) using the following protocol: 10 min 95 °C (95 °C 0:15 min, 57 °C 0:30 min+plate read, 72 °C 0:30 min) ×49, 72 °C 5 min 25 °C 0:10 min. Data were analyzed using the 2^-dCT method.

RNA Isolation

For this, 100–300 mg of feces were dissolved in 1 ml of lysis buffer and 40 µL of Proteinase K with vortexing. Samples were heated for 5 min at 95 °C. After 2 min of cooling at RT and 1 min of vortexing, samples were incubated for 5 min at 56 °C and then centrifuged for 5 min (<10.000× *g*). Supernatant was taken, and DNA was isolated with the Maxwell RSC Fecal Microbiome DNA Kit. Used primer sequences see Table 1.

## 3. Results

### 3.1. Setting 1 (ASF Analysis in Controlled Infection in C57Bl/6J and Balb/c Mice)

**Figure 1:**
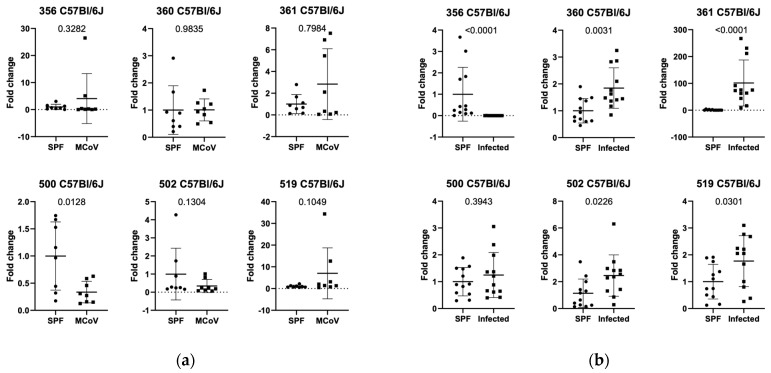
(**a**) Relative expression in MCoV—infected mice compared to control animals (SPF housing) in *C57Bl/6 J* mice and (**b**) relative expression of altered Schaedler flora in *C57Bl/6 J* mice (in-house) in SPF and quarantine areas. For numbering of ASF groups, see Table 2.

To understand the changes in the degree of alteration due to a virus infection in mice, we infected *C57Bl/6J* mice with *murine coronavirus* (MCoV) and determined the abundance of altered Schaedler flora (ASF) components in the feces of the animals. We observed that ASF 500 (*Pseudoflavonifactor* sp.) showed a significant decrease (*p* value 0.0128 in relative expression in MCoV-infected mice compared to control animals (Figure 1a). We were interested in whether these observations would occur only within an acute setting of a virus infection or if changes could also be observed in animals from controlled housing and naturally infected environments.

**Figure 2:**
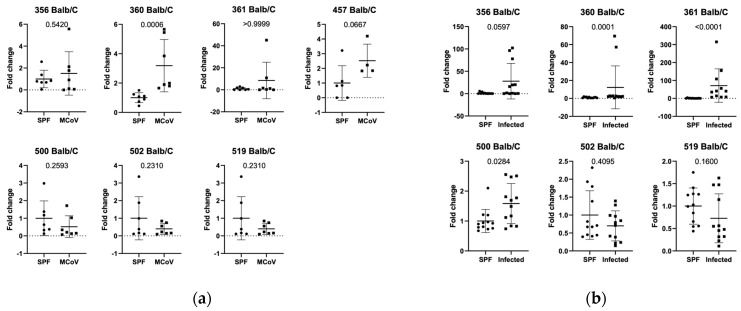
(**a**) Relative expression in MCoV—infected mice compared to control animals (SPF status) in Balb/c mice and (**b**) relative expression of altered Schaedler flora in Balb/c mice in SPF and quarantine areas in—house. For numbering of ASF groups, see Table 2.

Since differences in microbiome composition between *C57Bl/6J* and *Balb/c* mice have been described, ASF in *Balb/c* animals was also evaluated during MCoV infection. We observed a significant increase in *Lactobacillus intestinalis* (360, *p*-value 0.0006) after infection (Figure 2a).

### 3.2. Setting 2a (ASF Analysis in Natural Infection in C57Bl/6J Mice in Facility 1)

Analysis of ASF from *C57Bl/6J* mice in SPF (specific pathogen-free, according to FELASA guidelines [20]) and quarantine areas (confirmed infection of the area with FELASA-relevant pathogens [20]) revealed significant differences in the relative expression of ASF groups 356, 361, 502, and 519 (Figure 1b). ASF 356 (*p*-value < 0.0001, *Clostridium* sp.) in naturally infected *C57Bl/6J* mice showed a significant decrease compared to mice in SPF housing conditions. In contrast, ASF 360 (*Lactobacillus murinus*), 361 (*p*-value < 0.0001, *Lactobacillus murinus*), 502 (*p*-value 0.0121, *Clostridium* sp.), and 519 (*p*-value 0.0332, *Parabacteroides goldsteinii*) showed a significantly increased relative expression compared to SPF-housed C57Bl/6J (Figure 1b).

**Figure 3:**
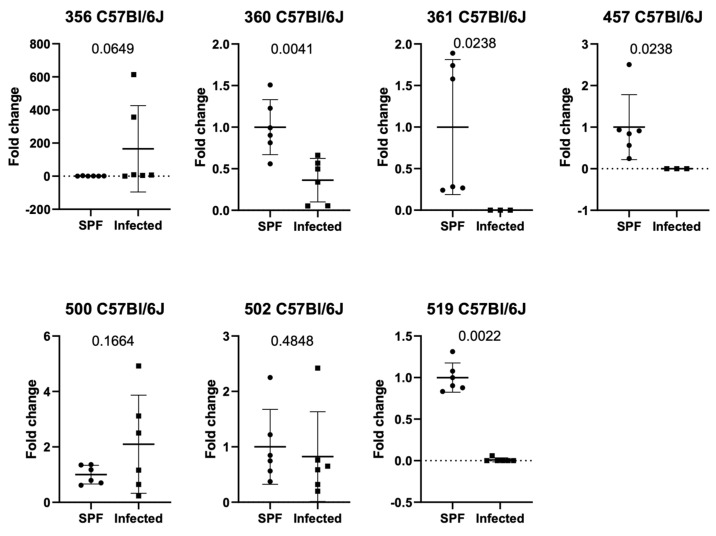
Relative expression of ASF in naturally infected mice compared to SPF *C57Bl/6 J* mice in an external facility. For numbering of ASF groups, see Table 2.

To extend existing results and evaluate if the observations could be confirmed at a different animal facility, fecal samples from *C57Bl/6J* mice with natural infection vs. no infection from an independent animal house were analyzed using the same methods. The relative expression of ASF 360 (*p*-value 0.0087), 361 (*p*-value 0.0238), 457 (*p*-value 0.0238, *Mucispirillum schaedleri*), and 519 (*p*-value 0.0022) showed a significant decrease in naturally infected mice (confirmed infection with FELASA-relevant pathogens [20]) compared to SPF-housed mice (Figure 3).

### 3.3. Setting 2b (ASF Analysis in Natural Infection in Balb/c Mice in Facility 1)

Similar to *C57Bl/6J*, we evaluated if Balb/c mice from specific pathogen-free (SPF) and quarantine units showed changes in ASF and if those changes were observable during natural pathogen challenges. We observed increased DNA amounts in the feces of ASF 356 (*p*-value 0.0597), 361 (*p*-value < 0.0001), and 500 (*p*-value 0.0284) (Figure 2b).

### 3.4. Setting 3 (ASF Analysis in Balb/c and C57Bl/6J in Natural Infection in Facility 2)

**Figure 4:**
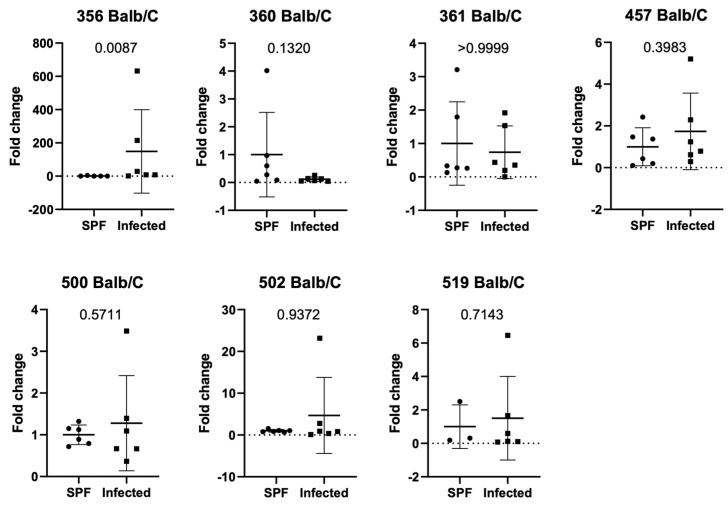
Relative expression of ASF in Balb/c mice in naturally infected mice compared to SPF—housed mice in an external facility. For numbering ASF groups, see Table 2.

To confirm our data generated within the same institution in an independently managed environment, we also analyzed *Balb/c* animals from an external husbandry for differences in ASF between SPF and quarantine units (Figure 4). We found that ASF 356 was increased (*p*-value 0.0087) in animals from quarantine, indicating a different ASF profile under natural infection in a different mouse facility.

### 3.5. Additional Findings (ASF Analysis in SPF Balb/c and C57Bl/6J in Facility 1 and 2)

**Figure 5:**
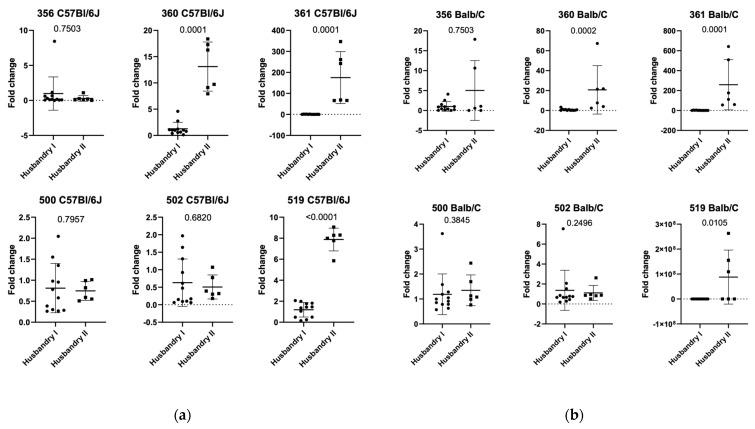
(**a**) Comparison of relative expression of ASF in *C57Bl/6 J* in SPF area in—house (Husbandry I) and external facility (Husbandry II) and (**b**) comparison of relative expression of *Balb/c* in SPF area in-house (Husbandry I) and external facility (Husbandry II). For numbering of ASF groups, see Table 2.

Furthermore, we were interested to see if there were differences in the relative expression of ASF flora between the two husbandries of *C57Bl/6J* and *Balb/c* mice housed in SPF conditions (see Figure 5a,b). Comparing SPF *C57Bl/6J* between husbandry 1 and 2, our data show a significant increase in ASF 360, 361, and 519. Comparing SPF *Balb/c* mice between Husbandry 1 and 2, a significant decrease in ASF 360, 361, and 519 was seen.

## 4. Discussion

Bacterial groups of ASF were analyzed in three different settings. The results clearly show a difference between infections with FELASA-relevant pathogens (controlled and natural infection) [20] and mice that lacked any of these pathogens. In this experimental setup, viral infections were predominant and showed a significant change in the quantity of Altered Schaedler Flora. A few cases have been described in the literature to date in which the microbiome impacts symptomatic and histological changes caused by infectious pathogens [22,23]. However, there is little evidence of the extent to which ASF itself changes due to pathogen contact. For example, some pathogens compete with ASF bacteria for nutrients, adhesion sites, and space in the gut or produce antimicrobial compounds or toxins that selectively inhibit commensal bacteria [24]. A pathogen like Salmonella may outcompete ASF strains such as *Lactobacillus murinus* (ASF 361) for access to intestinal epithelial cells [25]. Therefore, the number detected may be higher in the case of infections. Infections also trigger inflammatory responses that alter the gut environment, such as pH changes, increased reactive oxygen species, or cytokine release. Therefore, inflammation can preferentially harm certain ASF strains, reducing their abundance [26]. For example, *Clostridium species* in ASF are sensitive to inflammatory conditions, leading to a decline in their population during infections like colitis [27]. With the knowledge that ASF also changes with pathogen contact, an infection in an animal housing facility can be detected.

Our results show a significant difference in the quantity of ASF strains when comparing SPF and quarantine husbandry units in all three settings and mouse strains (*C57Bl/6J, Balb/c*). While there is a consistent pattern of change, the diverse groups are altered differently depending on the setting, location, and strain. Before starting to evaluate ASF in mice harboring infectious pathogens, it is important to define a baseline, as we can confirm previously described strain- and facility-dependent differences in the composition of the ASF [14]. In comparison to *C57Bl/6J* mice ASF, group 500 (Pseudoflavonifractor sp.) was significantly increased during MHV infection, and a different ASF group (360-Lactobacillus intestinalis) showed significant contribution in *Balb/c* mice. *Balb/c* mice in SPF housing conditions demonstrated fewer significant variations in ASF groups than *C57Bl/6J* mice compared to spontaneous infection. ASF 361 is increased in both naturally infected *Balb/c* and *C57Bl/6J* mice. Furthermore, ASF 356 showed a difference in distribution within both strains as follows: In *C57Bl/6J*, the expression in naturally infected mice is decreased, but it is increased in naturally infected *Balb/c*. Comparing both strains in the external animal house, different ASF groups showed significant contributions.

Our data indicate that both mouse strains show differences in their ASF between SPF and quarantine units with proven infections. However, the ASF components that changed differed within the facilities. We therefore determined the difference in baseline ASF relative abundance in both facilities by analyzing only animals from the SPF unit. We found that within *C57Bl/6J*, ASF 360, ASF 361, and ASF 519 were all upregulated in the second facility (Figure 5a). Similarly, we found differences in ASF for *Balb/c* mice, with ASF 360 and ASF 361 being significantly different between the two facilities (Figure 5b).

As mentioned in the introduction section, ASF is influenced by various factors of the microenvironment, such as housing facility, hygiene status, genetics, diet, and cage mates [14]. The housing facilities investigated in this paper also used different diets (Husbandry 1: Altromin, Husbandry 2: Altromin and Sniff), which could also have an influence on the data obtained. Although the diet from Altromin and Sniff differs only slightly in its composition, it can have an impact on relative expression of ASF. As nearly every other laboratory animal housing facility uses different diets, it is important to define an in-house baseline of ASF to exclude diet as a possible influencing factor, as long as the diet is not changed. If this is the case, an in-house baseline must be defined again.

Various testing methods are currently used to assess the hygiene status of laboratory mice. Each laboratory animal facility can decide which methods to implement. The most common approaches include the use of soiled bedding sentinel mice (testing the entire animal via pathohistological examination, feces, and blood analysis), environmental health monitoring (EHM), which uses filters exposed to soiled bedding or integrated into ventilation systems, and direct animal swabbing (fur and oral swabs, as well as feces collection), among others [28].

Both EHM and direct health monitoring (DHM), including sentinel testing and swabbing, have distinct advantages and disadvantages. EHM offers early detection and high sensitivity, capable of identifying low levels of pathogens. However, it may also detect residual DNA from non-viable organisms, leading to false positives. Additionally, uneven pathogen distribution in the environment can result in false negatives [29]. In contrast, DHM provides detailed information about the health status of individual animals, including clinical signs, pathological changes, and immune responses. This allows for the detection of both known and emerging pathogens—information that remains essential for ensuring the quality of laboratory animal research.

Integrating both methods can provide a comprehensive overview of colony health by combining the detailed insights of DHM with the ethical and sensitive detection capabilities of EHM. The choice of method should be guided by the specific research context, ethical considerations, and available resources. Analyzing the Altered Schaedler Flora (ASF) may be seen as a missing link between EHM and DHM, offering a hybrid solution for evaluating the health status of laboratory animals.

ASF analysis can be performed using qPCR kits (see Section 2, pp. 3–4) and does not require microbiome sequencing. A key advantage is that samples can be analyzed in-house without the need for external laboratories, as is often necessary in breeding facilities, allowing for prompt results. A baseline ASF profile must be established in an infection-free facility. For routine hygiene screening, these predefined ASF groups can then be analyzed internally using the described methods. If significant increases or decreases in specific ASF bacterial groups are observed, samples can be sent to external laboratories for further pathogen detection. In this context, the Altered Schaedler Flora functions as a sentinel flora that may help detect the presence of FELASA-relevant pathogens and provide valuable insights into overall health status.

This study is limited by its small sample size and retrospective design, which may restrict the generalizability of the findings. To validate and strengthen these initial observations, future studies should be conducted in larger, controlled settings with repeated experiments. A prospective approach encompassing the entire animal facility would allow for more robust conclusions and improved reproducibility. As a future perspective, such a comprehensive and standardized study design will be essential to fully understand the biological relevance and translational potential of our findings.

## 5. Conclusions

In this study we investigated the impact of natural and controlled infections with detected pathogens on defined gut bacterial groups (ASF-Altered Schaedler Flora) composition using two different mouse strains (*C57Bl/6J* and *Balb/c* mice). *C57Bl/6J* mice infected with MCoV showed a decrease in ASF 500 (Pseudoflavonifactor sp.) compared to controls (Figure 1a). Analysis of ASF in specific pathogen-free (SPF) and naturally infected *C57Bl/6J* mice revealed significant differences in ASF groups 356, 361, 502, and 519, with ASF 356 reduced and others increased in naturally infected mice (Figure 1b). In an independent facility, naturally infected *C57Bl/6J* mice exhibited decreased ASF 360, 361, and 457 compared to SPF mice (Figure 2). Similar ASF changes were found in *Balb/c* mice during MCoV infection, with increased Lactobacillus intestinalis (ASF 360) (Figure 3 and Figure 4). These findings were consistent across different facilities, confirming ASF profile variations under natural infections (Figure 5a,b).

## Figures and Tables

**Table 1:** Used primer sequences.

Target	Species	Fwd-Seq.	Rev-Seq.
356	*Clostridium* sp.	AAA ATA ATT AGG AGC TTG CTT TTA A	TTA GAA GAT GCC TCC TAA GAA CC
360	*Lactobacillus intestinalis*	GGT GAT GAC GCT GGG AAC	AAG CAA TAG CCA TGC AGC
316	*Lactobacillus murinus*	GAA CGA AAC TTC TTT ATC ACC	TAG CAT AGC CAC CTT TTA CA
457	*Mucispirillum schaedleri*	TCT CTT CGG GGA TGA TTA AAC	AAC TTT TCC TAT ATA AAC ATG CAC
492	*Eubacterium plexicaudatum*	AAT TCC TTC GGG GAG GAA GC	TAA AAC CAT GCG GTT TTA AAA AC
500	*Pseudoflavonifractor* sp.	ACG GAG GAC CCC TGA AGG	AGC GAT AAA TCT TTG ATG TCC
502	*Clostridium* sp.	GAG CGA AGC ACT TTT TTA GAA C	TTA CAC CAC CTC AGT TTT TAC C
519	*Parabacteroides goldsteinii*	GCA GCA CGA TGT AGC AAT ACA	TTA ACA AAT ATT TCC ATG TGG AAC
515R	Universal 16S		TTACCGCGGCTGCTGGCAC
8F	Universal 16S	CTC CTA CGG GAG GCA GCA G	

**Table 2:** Overview ASF bacterial groups with respective numbering.

ASF Group	Group Name
356	*Clostridium* sp.
360	*Lactobacillus intestinalis*
361	*Lactobacillus murinus*
457	*Mucispirillum schaedleri*
492	*Eubacterium plexicaudatum*
500	*Pseudoflavonifractor* sp.
502	*Clostridium* sp.
519	*Parabacteroides goldsteinii*

## Data Availability

The original contributions presented in this study are included in the article. Further inquiries can be directed to the corresponding author.
